# Supplementary figures and images for: Live births from urine derived cells
Source: PLoS One. 2023 Jan 25;18(1):e0278607. doi: 10.1371/journal.pone.0278607 (PMC9876353; doi:10.1371/journal.pone.0278607)

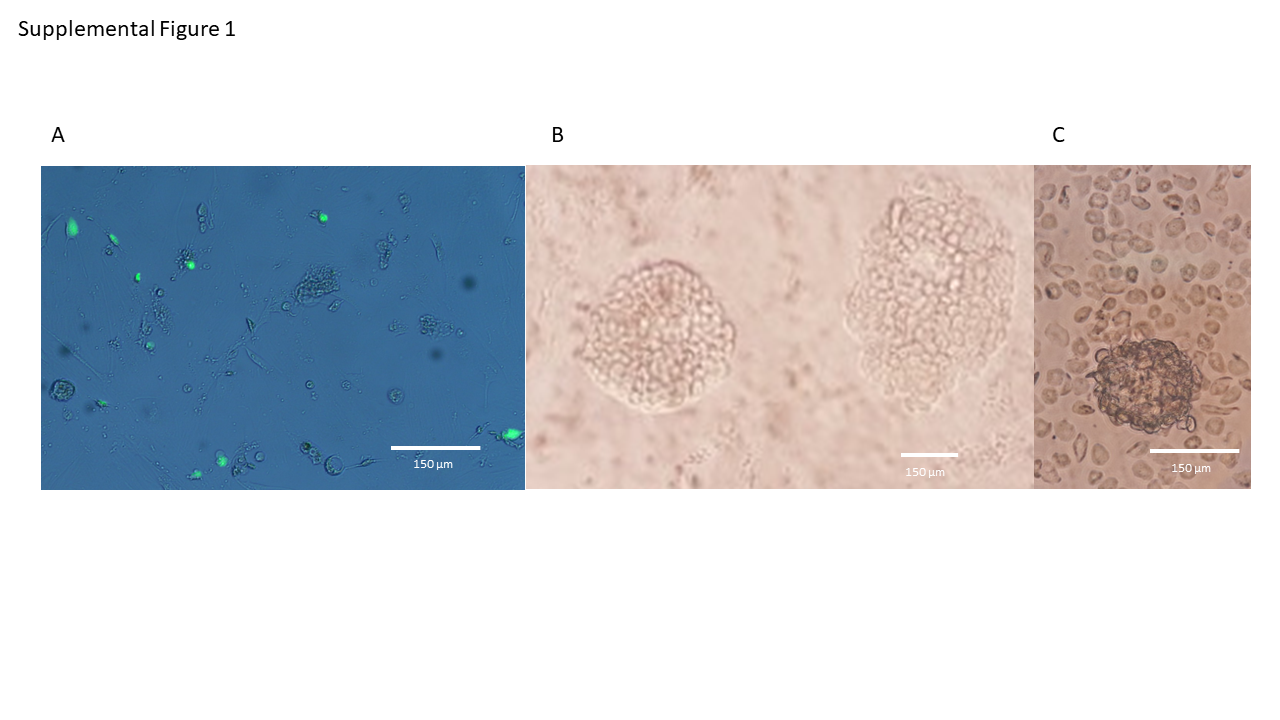

Supplement: S1 Fig — A) co-transfected canine UDCs with an iPSC inducing plasmid and expressing GFP (green) and UDCs morphological change 48hrs following transfection. B) embryoid body like formation C) senescent cell morphology and embyroid body like structure 10 days following iPSC plasmid transfection. (TIF) [file pone.0278607.s001.tif]
