# Supplementary material for: Live births from urine derived cells
Source: PLoS One. 2023 Jan 25;18(1):e0278607. doi: 10.1371/journal.pone.0278607 (PMC9876353; doi:10.1371/journal.pone.0278607)
Supplement: S1 Table — Effectivity compared with the same conditions, replicates of 3 or more were done in each case using the same urine source and cell number, effectivity represents observational differences alone rated “+” beneficial, “n” = neutral or effect not noticeably different than the control and “-” representing negative or potentially negative effects. *-/+ concentration dependent, high concentration negative effect, lower concentration positive effect. (DOCX) [file pone.0278607.s002.docx]

Supplemental Table 1.

| Observations in UDC establishment | | | | | |  |
| --- | --- | --- | --- | --- | --- | --- |
|  |  |  |  | | | |
| Culture Additions |  |  | Attachment | Growth | Increased Effectivity | |
| **Plate coatings** | |  |  |  |  | |
| Collagen | |  | Yes | Yes | n | |
| Collagen + Laminin | | | Yes | Yes | + | |
| Laminin | | | Yes | Yes | + | |
| Vitronectin/ | | | Yes | Yes | n | |
| Fibronectin BSA |  |  | Yes | Yes | - | |
|  | | |  |  |  | |
| **Urine** (sterile filtered) | | | Yes | Yes | n | |
| EGF | | | Yes | Yes | + | |
| EGF+insulin | | |  | 1 | -/+ | |
| Hydrocortisone | | | Yes | Yes | -/+ | |
| bFGF | | | No | Yes | n | |
| bFGF+insulin | | | Yes | Moderate | n | |
| Insulin | | | Yes | Moderate | - | |
|  | | |  |  |  | |

Supplemental Table 1.)

UDC culture conditions and result of culture.
Effectivity compared with the same conditions, replicates of 3 or more were done in each case using the same urine source and cell number, effectivity represents observational differences alone rated “+” beneficial, “n” = neutral or effect not noticeably different than the control and “-” representing negative or potentially negative effects. *-/+ concentration dependent, high concentration negative effect, lower concentration positive effect.
