# Supplementary material for: Live births from urine derived cells
Source: PLoS One. 2023 Jan 25;18(1):e0278607. doi: 10.1371/journal.pone.0278607 (PMC9876353; doi:10.1371/journal.pone.0278607)
Supplement: Attachment — Submitted filename: Reviewer Responses May 2022.docx [file pone.0278607.s003.docx]

PONE-D-22-06002

Dogs birthed from urine: A noninvasive source of cells for reproductive cloning and other technologies

PLOS ONE

Dear Dr. Olsson,

Thank you for submitting your manuscript to PLOS ONE. After careful consideration, we feel that it has merit but does not fully meet PLOS ONE’s publication criteria as it currently stands. Therefore, we invite you to submit a revised version of the manuscript that addresses the points raised during the review process.

For quite sometime, I was unable to secure a second reviewer who would accept the reviewing duty.  Therefore I served as the second reviewer.  After making comments on just a few pages, I realized that without page/line numbers, the task of review is very confusing.  I therefore did not finish the first round of review.  Please address comments from Reviewer 1 and my limited comments from the first few pages.  I will be able to finish the first round of review after page/line numbers are added.

Thank you very much for the effort and for the review. I will address you and the second reviewers concerns, make the adjustments in the text and provide a reference and response in-line and in blue for the comments below.

Please see my comments below:

There are no line or page numbers.  Need to add them.
Both line and page numbers have been added.

Abstract: cells can be obtained from genetically important or infertile animals without using urine.  The use of urine cell is not well justified.  The statement needs re-written.

 This has been addressed further in the text.

Remove UCD and UDC, there are many inconsistencies and are confusing, just use urine cells.

The UCD typo was corrected, thank you for pointing it out.
There is an important distinction between Urine derived Cells (UDCs) and urine cells. Urine cells are present in the urine and not cultured, but cells derived from urine, UDCs, are cultured and expandable, they can be saved, preserved and used for multiple applications, which differs greatly from urine cells alone, which may or may not be viable, expandable and may have different degrees of integrity.

Introduction: references are preceded with sentence stop “.”.  the stop should be placed after the references.  References separated from the preceding word with a space. This should be changed throughout the manuscript.

 This was changed in the introduction as well in the rest of the text were the space was added, this inconsistency was overlooked by us, thank you for noticing it.

What are sensitive animals?

Sensitive animals: in this context would be sensitive to methods where cells are taken, this nearly exclusively entails a biopsy, even in the case where blood must be taken animals are restrained. In the case of urine, behavioral collection methods can be employed to avoid any stress on “sensitive” animals.

For cell sampling, the authors only discussed tissue biopsy, blood samples are not discussed.

We did briefly address this lines 188 and 214, and references 25,39 and 40. This is because of general practice and due to the nature of adherent cell culture. Generally, cells cultured from blood are non-adherent, and there are a number of cell types such as lymphocytes, eosinophils and macrophages but they have culture limitations compared to adherent cell types.

“Cell type and passage” the sentence needs re-written.

This has been re-written as follows: Cell type and passage number have been implicated as effectors of cloning efficiency, with cumulus cells, stem cells and embryonic cells being more efficient in producing offspring, lacking abnormalities, than terminally differentiated

The sentence “To determine” needs to be re-written.
We updated the To determine sentence in line 65 to be more grammatically understandable:

The to determine on line 135 was likewise adjusted to simplify the understanding.

line 144 we added UDC and fibroblast derived (to clarify the source if it was not understood from the text)

Mitochondria from donor cells have been shown to be present in cloned offspring. Citing one paper that did not find them is not appropriate here.

Additional reference from Takeda et al 2013 added which describes the mitochondrial transfer and effect in cloning in inter and intra-species cloning. It is also true that there is more information needed on this

Remove “employed”.
Removed line 78

Please submit your revised manuscript by Jun 06 2022 11:59PM. If you will need more time than this to complete your revisions, please reply to this message or contact the journal office at [plosone@plos.org](mailto:plosone@plos.org). When you're ready to submit your revision, log on to <https://www.editorialmanager.com/pone/> and select the 'Submissions Needing Revision' folder to locate your manuscript file.

Please include the following items when submitting your revised manuscript:

- A rebuttal letter that responds to each point raised by the academic editor and reviewer(s). You should upload this letter as a separate file labeled 'Response to Reviewers'.
  (this document)
- A marked-up copy of your manuscript that highlights changes made to the original version. You should upload this as a separate file labeled 'Revised Manuscript with Track Changes'.
  Attached
- An unmarked version of your revised paper without tracked changes. You should upload this as a separate file labeled 'Manuscript'.
  Attached

If you would like to make changes to your financial disclosure, please include your updated statement in your cover letter. Guidelines for resubmitting your figure files are available below the reviewer comments at the end of this letter.

If applicable, we recommend that you deposit your laboratory protocols in [protocols.io](http://protocols.io/) to enhance the reproducibility of your results. Protocols.io assigns your protocol its own identifier (DOI) so that it can be cited independently in the future. For instructions see: <https://journals.plos.org/plosone/s/submission-guidelines#loc-laboratory-protocols>. Additionally, PLOS ONE offers an option for publishing peer-reviewed Lab Protocol articles, which describe protocols hosted on [protocols.io](http://protocols.io/). Read more information on sharing protocols at <https://plos.org/protocols?utm_medium=editorial-email&utm_source=authorletters&utm_campaign=protocols>.

We look forward to receiving your revised manuscript.

Kind regards,

Xiuchun Tian
Academic Editor
PLOS ONE

Journal Requirements:

When submitting your revision, we need you to address these additional requirements.

1. Please ensure that your manuscript meets PLOS ONE's style requirements, including those for file naming. The PLOS ONE style templates can be found at

<https://journals.plos.org/plosone/s/file?id=wjVg/PLOSOne_formatting_sample_main_body.pdf> and

<https://journals.plos.org/plosone/s/file?id=ba62/PLOSOne_formatting_sample_title_authors_affiliations.pdf>

2. Thank you for stating the following financial disclosure:

“This work was supported in part by the Korea Institute of Planning and Evaluation for Technology in Food, Agriculture, Forestry and Fisheries (IPET), through the Agri-Bio Industry Technology Development Program, funded by the Ministry of Agriculture, Food and Rural Affairs (MAFRA; grant number: 318016-5).”

Please state what role the funders took in the study.  If the funders had no role, please state: "The funders had no role in study design, data collection and analysis, decision to publish, or preparation of the manuscript."

If this statement is not correct you must amend it as needed.

Please include this amended Role of Funder statement in your cover letter; we will change the online submission form on your behalf.

3. We note that you have indicated that data from this study are available upon request. PLOS only allows data to be available upon request if there are legal or ethical restrictions on sharing data publicly. For more information on unacceptable data access restrictions, please see <http://journals.plos.org/plosone/s/data-availability#loc-unacceptable-data-access-restrictions>.

In your revised cover letter, please address the following prompts:

a) If there are ethical or legal restrictions on sharing a de-identified data set, please explain them in detail (e.g., data contain potentially sensitive information, data are owned by a third-party organization, etc.) and who has imposed them (e.g., an ethics committee). Please also provide contact information for a data access committee, ethics committee, or other institutional body to which data requests may be sent.

b) If there are no restrictions, please upload the minimal anonymized data set necessary to replicate your study findings as either Supporting Information files or to a stable, public repository and provide us with the relevant URLs, DOIs, or accession numbers. For a list of acceptable repositories, please see <http://journals.plos.org/plosone/s/data-availability#loc-recommended-repositories>.

We will update your Data Availability statement on your behalf to reflect the information you provide.

4. We note that you have included the phrase “data not shown” in your manuscript. Unfortunately, this does not meet our data sharing requirements. PLOS does not permit references to inaccessible data. We require that authors provide all relevant data within the paper, Supporting Information files, or in an acceptable, public repository. Please add a citation to support this phrase or upload the data that corresponds with these findings to a stable repository (such as Figshare or Dryad) and provide and URLs, DOIs, or accession numbers that may be used to access these data. Or, if the data are not a core part of the research being presented in your study, we ask that you remove the phrase that refers to these data.
Corrected

5. Please include a copy of Table 2 which you refer to in your text on page 7.
Addressed, this was an improper reference to
This was corrected in the text with (table 2 removed) The tables were previously merged and this data presented in the text alone

6. Please include captions for your Supporting Information files at the end of your manuscript, and update any in-text citations to match accordingly. Please see our Supporting Information guidelines for more information: <http://journals.plos.org/plosone/s/supporting-information>.

Included line 666 and below

7. PLOS ONE now requires that authors provide the original uncropped and unadjusted images underlying all blot or gel results reported in a submission’s figures or Supporting Information files. This policy and the journal’s other requirements for blot/gel reporting and figure preparation are described in detail at <https://journals.plos.org/plosone/s/figures#loc-blot-and-gel-reporting-requirements> and <https://journals.plos.org/plosone/s/figures#loc-preparing-figures-from-image-files>. When you submit your revised manuscript, please ensure that your figures adhere fully to these guidelines and provide the original underlying images for all blot or gel data reported in your submission. See the following link for instructions on providing the original image data: <https://journals.plos.org/plosone/s/figures#loc-original-images-for-blots-and-gels>.

In your cover letter, please note whether your blot/gel image data are in Supporting Information or posted at a public data repository, provide the repository URL if relevant, and provide specific details as to which raw blot/gel images, if any, are not available. Email us at [plosone@plos.org](mailto:plosone@plos.org) if you have any questions.

addressed in cover letter
