# Supplementary material for: Live births from urine derived cells
Source: PLoS One. 2023 Jan 25;18(1):e0278607. doi: 10.1371/journal.pone.0278607 (PMC9876353; doi:10.1371/journal.pone.0278607)
Supplement: Attachment — Submitted filename: Response Oct 2022.docx [file pone.0278607.s004.docx]

ONE-D-22-06002R1

Live births from urine: A noninvasive source of cells for reproductive cloning and other technologies

PLOS ONE

Dear Dr. Olsson,

Thank you for submitting your manuscript to PLOS ONE. After careful consideration, we feel that it has merit but does not fully meet PLOS ONE’s publication criteria as it currently stands. Therefore, we invite you to submit a revised version of the manuscript that addresses the points raised during the review process.  

We’ve made a revision according to the suggestions below. Please find inline comments towards the suggestions in blue

The revised version is still in very poorly prepared form.  The manuscript is full of inconsistencies and errors such as abbreviations (hour, hrs), stain names, bold/fonts of section headings, expression of exponentials, space missing between numbers and their units, before brackets, missing part of a figure legend, missing punctuations.

Spacing has been done through the manuscript and hours have been shorted to hrs to be consistent per the request. It was not clear what as ment by stain name, the section headings and names are ready to be simply modified per specific request, as weve followed the template. We believe weve caught most of the items missed before and appreciate the continuation.


These have been rectified wherever found.

A thorough professional editing on English and writing is REQUIRED.

Below, I listed a number of changes that need to be made.  These are by no means the only problems.  The sub-standard preparation prevented me from going through the entire manuscript due to the sheer number of English and composition issues.  It is the job of the professional editing service and the authors to ensure that the writing is at the level scientific publication before submission.

Abstract: there was no mention of birth and pregnancy, and too much on cells and culture. We’ve included the pregnancy and birth numbers for canines in the abstract. The focus was weighted towards cellular survival and characteristics compared to births, which showed overall success, considering the potential future applications.

Remove UDC throughout the manuscript as required from the first round of review.

With respect what should this be replaced with? The cells are urine derived cells, we have shown that they are from multiple cell types. They are not urine cells alone, because they have been cultured and thus represent a likely independent population.

Introduction:

L42: replace one of the “range” with another word.
Done

L43: “modeling” is not the right term here.

modified

L52: remove “to obtain a tissue sample”

removed

L53-54: remove one of the “further”

removed

Please add in Introduction: animals of very endangered species may not be captured yet their urine may be obtained.

added

L56 and 57: needs re-written. The two issues before and after “and” are not equivalent.  The second one is particularly unrelated to “the production”.

simplified

L61: add a space before [7].
Added, thank you.

L66-68: sentence grammatically incorrect.

Modified and restructured

L68-71: as it was pointed out already in the first round of review, mtDNA from donor cells has been found to remain in the SCNT embryos and progenies.  Please change and add more references.

These 2 sentences were removed so as not to misrepresent the information. We have worked extensively and tested multiples independently finding no mtDNA from host cells in the clones we have produced. It is true that this may not always be the case, but to avoid any misunderstanding weve simply removed the statements.

L71-73: please remove one of the “tool”.  Also, what are “these tools”?  The preceding statement was about mitochondria.  In this entire manuscript, a common problem in the writing is that the sentences jumped around in sequences.  There is widespread lack of logical progression or connection. 

We’ve gone back through the manuscript to correct some of these and to make more clear the logical connections.

Results:

L92: Please mention the supplements. Please see the text “…supplemented with sterile filtered urine…”

L93: please change “+/-“ to its publishable format throughout the manuscript.  Remove “S.D”.
Removed

L96: what are the criteria of “small” and “large”? Please see the figure. Large cellsa re known as

Not sure what is meant by this, question as it was a bit incomplete. In the figures the cells are clearly visibly larger than the norm. No specific criteria were given, the Large cells are generally known to be immune associated cells, we did not stain for this, the cells were non-viable in all samples. These can be mostly clearly seen in Fig 2.

L98-99: any number below 10 should be spelt out. 
Agreed, thank you

L100: please explain why you determined that Trypan Blue did not work for viability.
Trypan blue
This was because the stain was not clearly absent or present from all cells and there were additional artifacts which could not be excluded as cells or not, they may or may not have been dead cells, but the use of a specific dye enabled certainty. Figure 2 shows the clarity in the staining.

Discussion at more than 7.5 pages is too long, please shorten by half.
We agree and have edited it down.

L357: did you have 27 bovine or 10 bovine?

bovine n= 27 and canine n = 10, thus the (n=27 and n=10, respectively), We have separated this to make it more clear for you.

Table 1: under the column heading “transfer method”, you listed “1^st^ trial”, this is not a method.

We understand where this might be confusing. We have renamed them to 1^st^ and 2^nd^ control and the heading to Donor cells transferred to make clear that this transfer method was not a method per say.

Fig. 1.  The letter “C” is embedded in B, figure legend has grammatical errors, lack of proper labeling for both X- and Y-axes in C.
This was corrected. Labels for X and Y axes in C were also corrected and the labels modified to clarify.

Please submit your revised manuscript by Oct 06 2022 11:59PM. If you will need more time than this to complete your revisions, please reply to this message or contact the journal office at [plosone@plos.org](mailto:plosone@plos.org). When you're ready to submit your revision, log on to <https://www.editorialmanager.com/pone/> and select the 'Submissions Needing Revision' folder to locate your manuscript file.

Please include the following items when submitting your revised manuscript:

- A rebuttal letter that responds to each point raised by the academic editor and reviewer(s). You should upload this letter as a separate file labeled 'Response to Reviewers'.
- A marked-up copy of your manuscript that highlights changes made to the original version. You should upload this as a separate file labeled 'Revised Manuscript with Track Changes'.
- An unmarked version of your revised paper without tracked changes. You should upload this as a separate file labeled 'Manuscript'.

If you would like to make changes to your financial disclosure, please include your updated statement in your cover letter. Guidelines for resubmitting your figure files are available below the reviewer comments at the end of this letter.

If applicable, we recommend that you deposit your laboratory protocols in [protocols.io](http://protocols.io/) to enhance the reproducibility of your results. Protocols.io assigns your protocol its own identifier (DOI) so that it can be cited independently in the future. For instructions see: <https://journals.plos.org/plosone/s/submission-guidelines#loc-laboratory-protocols>. Additionally, PLOS ONE offers an option for publishing peer-reviewed Lab Protocol articles, which describe protocols hosted on [protocols.io](http://protocols.io/). Read more information on sharing protocols at <https://plos.org/protocols?utm_medium=editorial-email&utm_source=authorletters&utm_campaign=protocols>.

We look forward to receiving your revised manuscript.

Kind regards,

Xiuchun Tian
Academic Editor
PLOS ONE

[Note: HTML markup is below. Please do not edit.]

Reviewers' comments:

Reviewer's Responses to Questions

**Comments to the Author**

1. If the authors have adequately addressed your comments raised in a previous round of review and you feel that this manuscript is now acceptable for publication, you may indicate that here to bypass the “Comments to the Author” section, enter your conflict of interest statement in the “Confidential to Editor” section, and submit your "Accept" recommendation.

Reviewer #1: (No Response)

2. Is the manuscript technically sound, and do the data support the conclusions?

The manuscript must describe a technically sound piece of scientific research with data that supports the conclusions. Experiments must have been conducted rigorously, with appropriate controls, replication, and sample sizes. The conclusions must be drawn appropriately based on the data presented.

Reviewer #1: Yes

3. Has the statistical analysis been performed appropriately and rigorously?

Reviewer #1: Yes

4. Have the authors made all data underlying the findings in their manuscript fully available?

The [PLOS Data policy](http://www.plosone.org/static/policies.action#sharing) requires authors to make all data underlying the findings described in their manuscript fully available without restriction, with rare exception (please refer to the Data Availability Statement in the manuscript PDF file). The data should be provided as part of the manuscript or its supporting information, or deposited to a public repository. For example, in addition to summary statistics, the data points behind means, medians and variance measures should be available. If there are restrictions on publicly sharing data—e.g. participant privacy or use of data from a third party—those must be specified.

Reviewer #1: Yes

5. Is the manuscript presented in an intelligible fashion and written in standard English?

PLOS ONE does not copyedit accepted manuscripts, so the language in submitted articles must be clear, correct, and unambiguous. Any typographical or grammatical errors should be corrected at revision, so please note any specific errors here.

Reviewer #1: No

6. Review Comments to the Author

Please use the space provided to explain your answers to the questions above. You may also include additional comments for the author, including concerns about dual publication, research ethics, or publication ethics. (Please upload your review as an attachment if it exceeds 20,000 characters)

Reviewer #1: Significant editing is still required. The first part of the title still sounds a little odd. Maybe “Live births from urine derived cells:”. Discussion is long and the flow could be improved. It should be clear earlier in manuscript that for the canine SCNT, cloned donor animals were used.

Line 22-24: Split into two sentences so that it is clear that UDC were used in canine cloning (led to live births) and bovine SCNT (in vitro development only). This was split.
Line 29: add comma after 4C added
Line 30-32: awkward and unclear. Revise to make it clear that SCNT embryos regardless of donor cell (or species?) did not differ from IVF controls.

This has been clarified and expanded upon in the subsequent sentences.
Line 33: add apostrophe to species

Had just found this before seeing your suggestion. Thank you.

Line 35: delete comma after material Done

Line 46: change processes to process Done
Line 53: cellular based should be hyphenated hyphen added
Line 55: change life to “live” Thank you for that
Line 56: add “after cloning” after offspring added added
Line 57: add “s” to application; delete “Offspring from” and add “s” to produce added and deleted
Line 58: delete for the use in deleted
Line 61: delete s of aspects deleted
Line 65: delete comma after efficiencies deleted
Line 67: add comma after UDCs and before the ability; add SCNT before embryos
modified based on the other reviewers comment, removed to avoid mtDNA discussion as you also suggest below… comment to follow
Line 68: change induce to “produce” changed, better word choice
Line 68-71: you need a transition here to indicate the relevance of mitochondria. It doesn’t fit. Also, many studies have found heteroplasmy in cloned blastocysts and a low percentage in live offspring. A few examples:
- Burgstaller JP, Schinogl P, Dinnyes A, Müller M, Steinborn R. Mitochondrial DNA heteroplasmy in ovine fetuses and sheep cloned by somatic cell nuclear transfer. BMC Dev Biol. 2007 Dec 21;7:141. doi: 10.1186/1471-213X-7-141. PMID: 18154666; PMCID: PMC2323970.
- Takeda K, Tasai M, Iwamoto M, Akita T, Tagami T, Nirasawa K, Hanada H, Onishi A. Transmission of mitochondrial DNA in pigs and progeny derived from nuclear transfer of Meishan pig fibroblast cells. Mol Reprod Dev. 2006 Mar;73(3):306-12. doi: 10.1002/mrd.20403. PMID: 16245357.
- Inoue K, Ogonuki N, Yamamoto Y, Takano K, Miki H, Mochida K, Ogura A. Tissue-specific distribution of donor mitochondrial DNA in cloned mice produced by somatic cell nuclear transfer. Genesis. 2004 Jun;39(2):79-83. doi: 10.1002/gene.20029. PMID: 15170692.

We removed these sentences. Yes heteroplasmic mtDNA in the cytoplasm has been observed, ever one that we have personally tested in adults have not shown this heteroplasmy additionally there are several studies which indicate a level of competition and that one mitochondrial group is expunged or out competed. Every incidence Ive seen of offspring from clones have not had this heteroplasmy continue and the oocyte donor’s mtDNA appears to be established over that contributed by the cell donor. There is some discussion how to overcome this as the mtDNA is seen in some conservation and other circles as a negative to the use of cloning technologies in conservation.

Line 71: change Together these tools to “SCNT may prove” since you are discussing SCNT technology in your introduction. This has been modified to reflect your comment as well as to remove the use of tools, which was had confused the meaning to others.
Line 74: add a comma after “In this manuscript” change to to “into” modified changed
Line 80: change to “as a model for human disease” removed
Line 83: change of to “for” so that it reads “several limitations for culture of canine oocytes…” good suggestion, reads better.
Line 91” change cells to “cell” changed
Line 97: change and to “as they were” so it reads “predominantly in canine samples as they were almost absent from bovine samples. Changed.
Line 99: change cells to “cell” corrected
Line 102-106: sentence is too long, suggest breaking up
 Broke into several smaller sentences.
Line 106: add comma after 72 hrs added
Line 107: delete attempted deleted
Line 188: add semicolon before and comma after however “attachment; however,” added
Line 129: add period after ablated added
Line 134: delete comma after SCNT deleted
Line 135: add a sentence describing the animals the fibroblasts and UDCs were collected from. added
Line 136: delete (SCNT) as it was already used in introduction deleted
Line 138: add comma after one corrected
Line 142: add apostrophe to groups “ groups’ ” added
Line 144: This is unclear “72% +/- 21SD continued” revision is needed removed as you’re correct it isn’t needed, just extra information on oocyte recovery.
Line 147-150: sentence is unclear and awkward, revision is needed Revised.
Line 154: add comma after transfer added added
Line 162: change very to “vary” changed
Line 164: delete are; delete related to and change to “in” so it reads “and appear indistinguishable in their health, vigor and vitality…” changed
Line 167: change for to “of”; add comma after UDCs changed
Line 168: change comma to “and” changed
Line 169: stem cell like should be hyphenated “stem cell-like” modified
Line 170: add comma after bodies added
Line 171: change rational to rationale; clarify what rationale you are referring to here changed and added
Line 174: OCT4 should be italicized italicized
Line 178: delete the comma after source deleted
Line 180: hyphenate cell based “cell-based” hyphenated
Line 182-184: This sentence is unclear. Do you mean to say that it is the first report of using UDCs from a cloned animal to make other clones? Yes and a bit more, better clarifed
Line 191: delete comma after urine deleted
Line 192 and 193: delete (UDCs) because this abbreviation as already been used earlier. done
Line 197: add commas “, also to our knowledge” completed
Line 198: add semicolon and delete to our knowledge so that it reads “UDCs; the only other report of cloning using UDCs…” much better
Line 201: delete s on modifications. This is unclear do you mean the methyltransferases that add H3K27me3 or genes regulated by this modification? It wasn’t clear to us either, just that there was a difference in the expression in genes which are known to regulate these modifications. The fact though that a differentitation difference may exist could explain a number of things, would need to look more at cell cycles too, opens more questions than answers.
Line 202-203: This sentence is unclear and needs revision. Revised to clarify
Line 213: delete include deleted
Line 215: delete from deleted
Line 215-220: discussion of the markers does not add much here. What’s the point?
Simplified and made more concise
Line 230: what stem cell component? Please elaborate. Maybe belongs with what you are trying to say at line 205?? Yes this is part of it, if we have a stem cell population which has some evidence
Line 239: where multiple breeds used? This is not outlined earlier and should be clarified in results. Only Saluki was mentioned in the methods and no info about bovine donor animals is present. Please correct in methods. Yes, although we only cloned the saluki multiple breeds and ages were used in both the cattle and canine samples. This was clarified in the methods.
Line 244: Figure X needs to be corrected Corrected Figure 2
Line 253-257: This sentence is long and awkward and needs revision. revised
Line 264: delete removed.
Line 288-292: resveratrol’s use in culture was not reported in results. Please check and revise. Removed completely as it doesn’t follow the story well enough, still quite interesting results, for a later time perhaps.
Line 307-308: change to resembled embryoid bodies; delete were observed. rectified
Line 315: delete methods deleted
Line 337-338: Sentence fragment. Revise. This was overlooked, now completed.
Line 340: delete comma after expansion deleted
Figure 2: no explanation of panel D in legend. Added

Very good review, Thank you.

7. PLOS authors have the option to publish the peer review history of their article ([what does this mean?](https://journals.plos.org/plosone/s/editorial-and-peer-review-process#loc-peer-review-history)). If published, this will include your full peer review and any attached files.


If you choose “no”, your identity will remain anonymous but your review may still be made public.


**Do you want your identity to be public for this peer review?** For information about this choice, including consent withdrawal, please see our [Privacy Policy](https://www.plos.org/privacy-policy).

Reviewer #1: No
